# Extracorporeal Cytokine Adsorption in Sepsis: Current Evidence and Future Perspectives

**DOI:** 10.3390/biomedicines13071684

**Published:** 2025-07-09

**Authors:** Matteo Guarino, Anna Costanzini, Francesco Luppi, Martina Maritati, Carlo Contini, Roberto De Giorgio, Michele Domenico Spampinato

**Affiliations:** 1 Department of Translational Medicine, St. Anna University Hospital of Ferrara, University of Ferrara, 44124 Ferrara, Italy; grnmtt@unife.it (M.G.); anna.costanzini@unife.it (A.C.); francesco.luppi@unife.it (F.L.); spmmhl@unife.it (M.D.S.); 2 Emergency Department, St. Anna University Hospital of Ferrara, 44124 Ferrara, Italy; 3 Infectious Diseases Unit, Department of Medical Sciences, University of Ferrara, 44124 Ferrara, Italy; martina.maritati@unife.it (M.M.); cnc@unife.it (C.C.)

**Keywords:** cytokine storm, extracorporeal cytokine adsorption, inflammation, mortality, sepsis, septic shock

## Abstract

**Background**: Sepsis and septic shock are major contributors to global morbidity and mortality. The “cytokine storm,” a hyper-inflammatory response, plays a central role in sepsis pathophysiology, leading to multi-organ failure. Extracorporeal cytokine adsorption therapies, such as CytoSorb, Toraymyxin, Oxiris, HA330/380, and Seraph 100 Microbind, aim to mitigate the inflammatory response by removing circulating cytokines and other mediators. **Methods**: A comprehensive search of Scopus and PubMed was conducted for studies published from January 2020 to May 2025. The search terms included “sepsis,” “septic shock,” and “extracorporeal cytokine adsorption.” Relevant studies, including clinical trials and meta-analyses, were included to assess the efficacy and safety of these therapies. **Results**: Extracorporeal cytokine adsorption has shown promising results in reducing cytokine levels, improving organ function, and decreasing vasopressor requirements. However, evidence regarding mortality reduction remains inconsistent. Studies have demonstrated benefits in sepsis, ARDS, and cardiogenic shock, improving organ recovery and inflammatory markers. **Conclusions**: Extracorporeal cytokine adsorption is a potential adjunctive therapy in sepsis management, offering improvements in organ function and inflammatory control. While the mortality benefit remains uncertain, ongoing research and large-scale clinical trials are essential to define its clinical role and optimize its application.

## 1. Introduction

Sepsis and septic shock represent critical global health challenges, accounting for significant morbidity and mortality worldwide [1,2]. Defined as a life-threatening organ dysfunction caused by a dysregulated host response to infection [3], sepsis progresses along a spectrum that includes an initial hyper-inflammatory phase, characterized by excessive cytokine release, commonly referred to as the “cytokine storm” [4]. This phenomenon is central to the pathophysiology of sepsis, contributing to endothelial dysfunction, tissue damage, and multi-organ failure [5]. Several strategies have been proposed to mitigate the cytokine storm in sepsis, including the use of neutralizing monoclonal antibodies, corticosteroids, and therapeutic plasma exchange. However, these approaches have shown variable efficacy and are often limited by timing, specificity, or adverse effects. Extracorporeal cytokine adsorption has emerged as a promising adjunctive therapy due to its ability to broadly reduce circulating inflammatory mediators in a non-pharmacological manner [1,6]. Without timely intervention, septic shock (which is marked by profound circulatory and metabolic abnormalities) can result in rapid patient deterioration and death [6].

Recent advances in our understanding of sepsis pathophysiology highlighted the complexity of the immune response, which typically evolves from an initial pro-inflammatory state to an immunosuppressive phase in many patients [5,7,8,9]. This dynamic change in the immunological landscape has driven the search for innovative therapeutic approaches that can modulate the host response, with the goal of mitigating the harmful effects of excessive inflammation while preserving the immune system’s ability to contrast infection. While cytokine adsorption is primarily aimed at controlling hyperinflammation, its role in the immunosuppressive phase of sepsis remains controversial. Some evidence suggests that early intervention may help restore immune balance before profound immune paralysis occurs, although timing and patient selection are critical [10,11,12,13,14,15].

Technologies such as CytoSorb [16], Toraymyxin [17], Oxiris [18], HA330/380 [19], and Seraph 100 Microbind [20] offer varying mechanisms to remove circulating cytokines, endotoxins, and other inflammatory mediators. These devices have been integrated into critical care settings worldwide, often in combination with renal replacement therapy or Extracorporeal Membrane Oxygenation (ECMO), to target the hyper-inflammatory state of septic/septic shock patients [12,21,22]. In addition to cytokines, endotoxins, particularly lipopolysaccharides (LPS) from Gram-negative bacteria, play a key role in sepsis pathogenesis by triggering Toll-like receptor-mediated inflammation [4]. Endotoxemia has been associated with worse outcomes in septic patients and specific adsorption devices, such as Toraymyxin, have been developed to target circulating endotoxins [17].

This narrative review has been thought to provide a comprehensive appraisal of extracorporeal cytokine adsorption in sepsis, focusing on the mechanisms, clinical evidence, limitations, and future directions of these technologies. By comparing their efficacy and safety profiles, we seek to delineate the current state-of-the-art and highlight potential pathways for optimizing clinical application of cytokine adsorption.

## 2. Search Strategy

A comprehensive search was conducted in Scopus and PubMed databases for studies published between January 2020 (the year CytoSorb was approved by the U.S. Food and Drug Administration [FDA]) and May 2025. The search terms included “sepsis” OR “septic shock” AND “adult” AND “extracorporeal cytokine adsorption” OR “CytoSorb” OR “Toraymyxin” OR “Oxiris” OR “HA330/380” OR “Seraph 100 Microbind”. In addition, a manual search of the reference lists from relevant studies and previous reviews was performed to further expand the scope of our analysis.

## 3. Pathophysiology of Cytokine Storm in Sepsis

**Figure 1:**
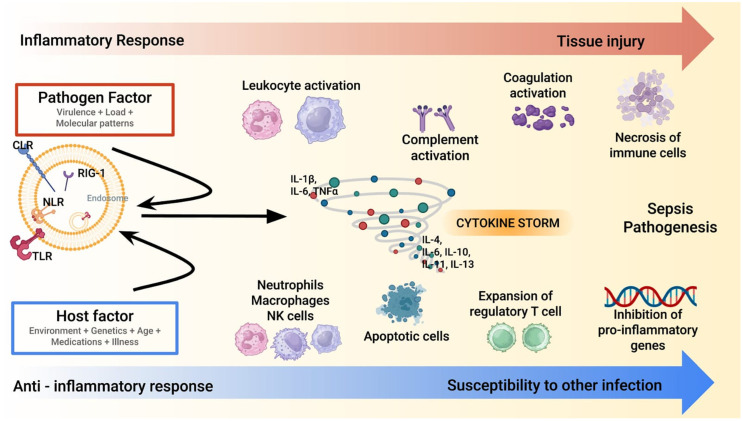
A synopsis of sepsis/septic shock pathophysiology. Sepsis pathogenesis involves a dysregulated immune response, where the pathogen and host factors trigger an excessive inflammatory cascade (red line). A cytokine storm amplifies leukocyte, complement, and coagulation activation, leading to tissue injury. Concurrently, an anti-inflammatory response (blue line) with regulatory T cell expansion and apoptotic immune cells increases susceptibility to secondary infections. *Note:* CLR: C-type Lectin Receptor; IL: Interleukin; NK: Natural Killer; NLR: Nod-like Receptor; RIG: Retinoic Acid-Inducible Gene-I-Like Receptors; TLR: Toll-like Receptors; TNF- α: Tumor Necrosis Factor-Alpha.

The cytokine storm in sepsis represents a hallmark of the host’s dysregulated immune response to infection [3,4,5,7,8,9]. This process is characterized by an overwhelming release of pro-inflammatory cytokines, such as tumor necrosis factor-α (TNF-α), interleukin-1β (IL-1β), and interleukin-6 (IL-6) (Figure 1).

These molecules act as primary mediators of systemic inflammation initiating a cascade of events, including endothelial activation, increased vascular permeability, and recruitment of immune cells to infection sites [23,24,25,26]. While these responses are initially protective, their excessive amplification can lead to widespread tissue injury and organ dysfunction. The hyper-inflammatory phase is often accompanied by disseminated intravascular coagulation (DIC), microvascular thrombosis, and mitochondrial dysfunction, which further exacerbate organ damage [27,28,29]. This stage is a critical target for interventions aimed at modulating the immune response to prevent irreversible multi-organ failure. As sepsis progresses, many patients evolve into an immunosuppressive phase characterized by decreased production of pro-inflammatory cytokines and impaired immune cell function. This phase is marked by lymphocyte apoptosis, monocyte dysfunction, and reduced expression of major histocompatibility complex class II (MHC-II) molecules. The resulting immune paralysis makes patients more susceptible to secondary infections, including opportunistic pathogens, complicating their clinical course. The interplay between these opposing immune states highlights the complexity of sepsis pathophysiology [4]. Although the cytokine storm model has historically guided therapeutic strategies, it is now recognized as an oversimplification of the immune dysregulation in sepsis. Not all patients exhibit markedly elevated cytokine levels, and the relationship between cytokine levels and clinical severity is largely inconsistent [4]. Moreover, this model does not fully account for other critical mechanisms of organ dysfunction, such as endothelial injury, mitochondrial impairment, and immune cell exhaustion. These limitations underscore the need for a broader immunological framework and support the development of more individualized approaches to extracorporeal therapies [4,5,7,8]. While the hyper-inflammatory phase drives initial organ damage, the subsequent immunosuppressive state poses a significant challenge for long-term recovery. Biomarkers such as interleukin-10 (IL-10), soluble programmed death-ligand 1 (sPD-L1), and monocyte human leukocyte antigen-DR (mHLA-DR) expression have been proposed to stratify patients and guide therapeutic interventions, although their routine clinical use remains limited [4,23].

Understanding the dual nature of the immune response in sepsis emphasizes the need for targeted therapies. Extracorporeal cytokine adsorption is one such approach, aiming at downsizing the harmful effects of cytokine overproduction during the hyper-inflammatory phase while preserving immune competence to effectively contrast infection [10]. In recent years, advances in sepsis research have led to the identification of distinct clinical and biological phenotypes, reflecting the heterogeneity of host responses [4,7,8]. These phenotypes (defined by combinations of inflammatory markers, organ dysfunction patterns, and clinical trajectories) include hyper-inflammatory, immunosuppressed, and mixed-response profiles. For instance, patients with elevated IL-6, TNF-α, and C-reactive protein (CRP) may represent a hyper-inflammatory phenotype, whereas those with low mHLA-DR expression and lymphopenia may exhibit features of immune-stunning paralysis [4,23]. Recognizing these phenotypes is crucial, as they may affect both the efficacy and timing of extracorporeal cytokine adsorption. Hyper-inflammatory patients may benefit from early cytokine removal, while immunosuppressed individuals could be at risk of further depletion of protective mediators [8]. Although current evidence remains limited, future studies should aim at stratifying patients based on immune profiles to personalize extracorporeal therapies and optimize clinical outcomes. This evolving understanding supports the hypothesis that extracorporeal cytokine adsorption may be beneficial not only during the hyper-inflammatory phase, by reducing excessive cytokine levels, but also in selected cases of immunosuppression, where the removal of inhibitory mediators could help restore immune competence. However, this dual-phase application remains to be validated in clinical studies [4,8,23].

## 4. Extracorporeal Cytokine Adsorption: Principles and Mechanisms

Extracorporeal cytokine adsorption has emerged as a promising therapeutic strategy to modulate the severe immune dysregulation associated with sepsis [12,13,14,15,16,17,18,19,20,21,22]. By removing pro-inflammatory mediators, endotoxins, and other circulating factors, this approach aims to reduce the inflammatory burden that drives the pathophysiology of sepsis [13,14]. Various extracorporeal devices, including CytoSorb, Toraymyxin, Oxiris, HA330/380, and Seraph 100 Microbind, have been developed with the goal of attenuating the cytokine storm in sepsis [16,17,18,19,20]. These devices use different mechanisms to target and remove inflammatory mediators and other harmful substances.

The underlying mechanisms of these devices can be broadly categorized into selective and non-selective adsorption. Selective adsorption devices, such as Toraymyxin and Seraph 100 [17,20], are designed to target specific molecules or pathogens, such as endotoxins or bacteria. Toraymyxin utilizes a polymyxin B-immobilized fiber membrane that binds to endotoxins, neutralizing their pro-inflammatory effects thus reducing their impact on the immune response [17,30,31,32,33,34]. Similarly, Seraph 100 Microbind is based on a filter that contains polymer beads coated with heparin, mimicking the human glycocalyx and acting as a binding site for pathogens. Hence, Seraph 100 Microbind selectively adsorbs endotoxins and some bacterial pathogens, helping to mitigate both microbial load and endotoxemia in sepsis [20,35,36]. Oxiris is a multifunctional hemofilter that combines high-flux dialysis with adsorption capabilities. It is composed of a polyacrylonitrile-based AN69 membrane surface-treated with polyethyleneimine (PEI), which enhances its ability to bind endotoxins and cytokines. Additionally, the membrane is heparin-coated to reduce thrombogenicity. Oxiris is designed to be used in continuous renal replacement therapy (CRRT) circuits, allowing for simultaneous renal support and immunomodulation. Its adsorption profile includes both pro-inflammatory cytokines (e.g., IL-6, TNF-α) and endotoxins, making it suitable for patients with septic shock and acute kidney injury [18,22]. HA330/380 cartridges are based on neutral macroporous resin adsorbents with a large surface area and pore size distribution optimized for middle-molecular-weight substances. These devices are non-selective and can remove a wide range of inflammatory mediators, including cytokines, Damage-Associated Molecular Patterns (DAMPs) and Pathogen-Associated Molecular Patterns (PAMPs) molecules. HA330/380 has been widely used in China and other Asian countries for sepsis, pancreatitis, and trauma-related systemic inflammation. Its mechanism relies on hydrophobic interactions and van der Waals forces to capture circulating toxins and inflammatory molecules [19].

In contrast to the selective mechanisms described above, non-selective adsorption devices (such as CytoSorb and HA330/380) are designed to remove a broader spectrum of circulating molecules based on their physicochemical characteristics, including size and hydrophobicity [16,19]. CytoSorb is a highly porous polymer-based filter of adsorbent resin composed of small spheres of porous polymeric material (polystyrene-divinylbenzene) covered with polyvinylpyrrolidone. This structure enables CytoSorb to adsorb hydrophobic molecules with a molecular weight between 5 and 55 KDa, to capture pro-inflammatory cytokines (e.g., TNF-α, IL-6, and IL-1β). While this approach effectively reduces systemic inflammation, it also poses the risk of indiscriminately remove both harmful and beneficial molecules, such as protective cytokines or coagulation factors [16,37,38,39,40,41]. This broad-spectrum removal can decrease the overall inflammatory burden, although a careful balance is required to avoid detrimental effects on the patient’s immune function [16,42]. Importantly, while CytoSorb effectively reduces pro-inflammatory cytokines, its non-selective adsorption profile may also lead to the removal of anti-inflammatory mediators, such as IL-10 and growth factors, involved in tissue repair. This unintended depletion could impair immune regulation and delay recovery, especially in patients evolving to an immunosuppressive phase [16,40,42].

Many cytokine adsorption devices are compatible with standard extracorporeal circuits (such as those used for renal replacement therapy or ECMO) enabling seamless integration into the critical care workflow. For instance, Oxiris and CytoSorb can be incorporated alongside CRRT [43]. This integration allows for simultaneous blood purification and cytokine removal, offering a comprehensive approach to manage both sepsis and acute kidney injury [12,44]. Additionally, devices like CytoSorb can be added to ECMO circuits to manage the inflammatory response in patients with severe respiratory or cardiac failure, without compromising oxygenation and perfusion [45].

The versatility of these technologies, along with their ability to be integrated into various extracorporeal modalities, highlights their potential in controlling the complex immune dysregulation occurring in sepsis [46,47]. However, careful consideration of patient-specific factors and the characteristic of each device is crucial to optimize therapeutic outcomes [48,49]. From a practical standpoint, extracorporeal cytokine adsorption is typically considered in patients with septic shock and high inflammatory burden, often reflected by elevated IL-6, CRP and/or endotoxin activity [16,40]. However, there is no universally accepted threshold to initiate therapy. The adequacy of adsorption is usually inferred from clinical improvement and biomarker trends, although standardized criteria are lacking [41]. In some cases, repeated sessions may be considered if cytokine levels rebound or clinical deterioration occurs [16]. Adverse effects include potential removal of antibiotics and beneficial mediators, highlighting the need for therapeutic drug monitoring and individualized treatment planning. Device selection depends on the clinical context: CytoSorb for broad cytokine removal [16], Toraymyxin for endotoxemia [17], and Seraph 100 for pathogen adsorption [20].

## 5. Extracorporeal Cytokine Adsorption Efficacy: Clinical Evidence

Research on extracorporeal cytokine adsorption in sepsis has led to an increasing body of clinical data supporting its efficacy. Several studies have evaluated the impact of cytokine adsorption on key outcomes, including mortality, cytokine levels, vasopressor use, and organ dysfunction. However, the results vary depending on the specific technology used, patient characteristics, and study methodologies.

*1. Sepsis and Septic Shock*

CytoSorb, one of the most extensively studied devices for extracorporeal cytokine adsorption, has shown promising results in various clinical trials. However, data regarding its impact on mortality are conflicting [16]. One key randomized trial evaluated the efficacy of CytoSorb in patients with sepsis and septic shock. This study reported a significant reduction in pro-inflammatory cytokines such as TNF-α, IL-6, and IL-1β, which are known to drive the cytokine storm in sepsis. While mortality was not consistently reduced, CytoSorb treatment led to the reduction in vasopressor therapy, suggesting potential modulation of vascular tone and improved tissue perfusion [50,51,52,53,54].

Other studies further confirmed that the use of CytoSorb resulted in decreased cytokine levels, with a significant improvement in the clinical status of patients, particularly in those with septic shock due to severe bacterial infections [16]. Despite this, mortality remains a challenging endpoint. Some studies report a reduction in mortality among patients treated with CytoSorb [53], while others show no significant benefits [50,55]. This variability may be attributed to differences in patient populations, severity of sepsis, and timing of treatment.

A crucial finding across these studies is the improvement of organ functions, which includes a reduction in acute kidney injury and hepatic dysfunction, potentially contributing to long-term survival [50]. Notably, the ability to reduce vasopressor requirements is a significant outcome. In several studies, there was a reduction in the use of norepinephrine and other vasopressors, suggesting that extracorporeal cytokine adsorption may have a direct effect on blood pressure regulation [51,52,53,54]. While the exact mechanisms are not fully understood, it is hypothesized that improved endothelial function and reduced inflammation can be contributing factors [54].

*2. Clinical Evidence for Other Adsorption Devices*

While CytoSorb remains the most extensively studied device in the field of extracorporeal cytokine adsorption, emerging clinical data have begun to shed light on the potential efficacy of other technologies. Among these, Toraymyxin (PMX) has been evaluated primarily in patients with intra-abdominal sepsis and high endotoxin activity. Several studies, including the EUPHAS trial, have reported an improvement of hemodynamic parameters and a reduction in vasopressor requirements following PMX hemoperfusion [17,18,19,20,21,22,23,24,25,26,27,28,29,30,31,32,33,34]. However, the impact on mortality remains controversial, with meta-analyses yielding mixed results and highlighting the need for more robust randomized controlled trials.

Oxiris, a multifunctional hemofilter used in CRRT, has gained attention for its dual capacity to remove both cytokines and endotoxins. Its integration into CRRT circuits allows for simultaneous renal support and immunomodulation, making it particularly suitable for patients with septic shock complicated by acute kidney injury [18]. A recent meta-analysis suggested a potential reduction in 28-day mortality and improved hemodynamic stability in patients treated with Oxiris, although high-quality evidence is still limited [22].

HA330 and HA380 cartridges, based on neutral macroporous resin technology, have been widely used in Asia for the treatment of sepsis and trauma-related systemic inflammation. These devices can adsorb a broad range of inflammatory mediators, including IL-6, TNF-α, and various DAMPs and PAMPs. Clinical studies have reported reductions in inflammatory markers and improvements in organ function, although most of the available data come from observational studies with limited external validation [19].

Finally, the Seraph 100 Microbind device represents a novel approach by targeting circulating pathogens and endotoxins through a heparin-coated surface that mimics the endothelial glycocalyx. Early clinical experiences suggest that Seraph 100 may contribute to improve bloodstream infections and reduced inflammatory burden in patients with septic shock. Importantly, pharmacokinetic studies indicate that Seraph 100 does not significantly adsorb most antibiotics, with the notable exception of aminoglycosides. This relative selectivity may represent a key advantage in preserving the efficacy of antimicrobial therapy during extracorporeal treatment [20].

Although the clinical evidence for these alternative devices is still evolving, their distinct mechanisms and potential applications in specific patient populations underscore the need for comparative studies and head-to-head trials. A more nuanced understanding of their efficacy, safety, and optimal indications will be essential to guide personalized treatment strategies in sepsis.

*3. Other Applications*

Extracorporeal cytokine adsorption has been applied in other severe clinical conditions, including acute respiratory distress syndrome (ARDS), cardiogenic shock, and cardiovascular surgery [12,21,22,39,56,57,58]. In these conditions, systemic inflammation plays a central role in clinical deterioration, and reducing pro-inflammatory mediators can lead to significant improvements.

ARDS is a common complication in patients with severe sepsis [1], and CytoSorb treatment has shown positive effects [39]. Several observational studies have highlighted a reduction in cytokine levels in these patients, with an improvement in respiratory function [39,59,60]. A 2021 study reported that CytoSorb was used in patients with severe ARDS associated with sepsis, revealing significant improvement in respiratory parameters, including the PaO_2_/FiO_2_ ratio, which is a key indicator of ARDS severity [39].

In cardiogenic shock—a form of acute circulatory failure due to heart dysfunction—systemic inflammation plays a critical role [56,58]. Treatment with CytoSorb in these patients has demonstrated a reduction in inflammatory biomarkers and a decrease in the incidence of multi-organ failure. Additionally, a study involving patients who underwent complex cardiovascular surgery showed improvements in postoperative recovery and a reduction in complications, suggesting that extracorporeal cytokine adsorption may have a protective effect for these individuals [61].

*4. Meta-analyses and Registries*

Meta-analyses and clinical registries have provided a comprehensive overview of the effects of extracorporeal cytokine adsorption therapies [16,62]. The COSMOS Registry, a multicenter registry collecting data from hospitals across several countries, is one of the most important sources of information. This registry has included thousands of patients treated with CytoSorb in various clinical settings, including sepsis, cardiogenic shock, and ARDS. The data suggest that CytoSorb use is associated with a reduction in pro-inflammatory cytokine levels and improvement in organ function, with a trend toward reduced mortality in patients with severe sepsis [63].

A 2022 meta-analysis examined over ten clinical trials involving CytoSorb and found a significant reduction in cytokine levels, such as IL-6 and TNF-α, in response to treatment. However, this study did not consistently demonstrate a reduction in mortality rates [62]. Nevertheless, improvements in organ function, including renal and hepatic function, were consistently observed across all studies included in the meta-analysis.

## 6. Limitations and Controversies

Despite the growing evidence supporting the use of extracorporeal cytokine adsorption in sepsis, several limitations and controversies still exist. One of the main challenges is the lack of definitive evidence regarding its impact on mortality. Although reductions in cytokine levels and improvements in organ function have been demonstrated, studies have failed to consistently show a clear benefit in terms of survival [50,55,62]. This inconsistency can be attributed to variations in study design, patient populations, and the timing of treatment, making it difficult to reach definitive conclusions about the mortality benefits of this therapy. Additionally, the risk of immunosuppression in the late stages of sepsis raises further concerns [48,64]. While early cytokine removal can help manage excessive inflammation, it may inadvertently suppress the immune system in advanced sepsis, leaving patients more susceptible to secondary infections [65]. Moreover, the non-selective nature of some adsorption devices may exacerbate this issue by removing protective cytokines and growth factors, such as IL-10 and Vascular Endothelial Growth Factor (VEGF), which play a role in immune resolution and tissue regeneration [16,40,42,48]. This highlights the need for more selective adsorption technologies and careful patient stratification. This raises questions about the balance between removing harmful mediators and preserving the immune response [66]. Furthermore, the use of extracorporeal cytokine adsorption requires a more personalized approach, as sepsis can present in different phenotypes, each with distinct inflammatory profiles.

Table 1 provides a detailed comparison of all the respective technologies and applications [17,19,22,34,40,67,68,69,70,71,72,73,74,75,76,77,78,79,80,81,82,83,84,85,86,87,88].

## 7. Future Directions

**Figure 2:**
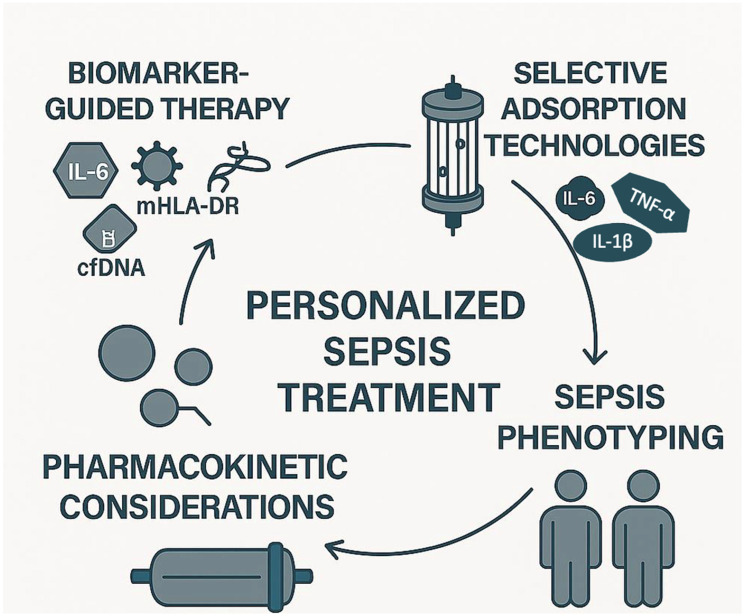
Future directions of extracorporeal cytokine adsorption in sepsis: towards personalized treatment. This infographic illustrates the four core elements driving the future of extracorporeal cytokine adsorption in sepsis management: (1) biomarker-guided therapy, using biomarkers such as IL-6, cfDNA, mHLA-DR, and LPS to identify immune states and guide treatment initiation; (2) selective adsorption technologies, designed to target specific inflammatory or anti-inflammatory mediators, thereby minimizing off-target effects; (3) sepsis phenotyping, enabling the stratification of patients based on immune profiles (e.g., hyper-inflammatory vs. immunosuppressed); and (4) pharmacokinetic considerations, to optimize therapy while avoiding unintended adsorption of essential medications. Together, these pillars support a precision medicine approach aiming to tailor extracorporeal therapy to individual patient needs. *Note:* Cell-free DNA; mHLA-DR: Monocyte human leukocyte antigen-DR; LPS: Lipopolysaccharide.

The future of extracorporeal cytokine adsorption in sepsis lies in several key areas of development (Figure 2).

First, the identification of reliable biomarkers, such as mHLA-DR expression and cytokine profiles, could enable more targeted therapies, allowing clinicians to select patients who would benefit most from this intervention. This personalized approach could optimize the timing and effectiveness of treatment. Among the most studied biomarkers, IL-6 is widely used to assess the inflammatory burden and monitor the response to treatment [16]. LPS levels, measured via endotoxin activity assays, are particularly relevant for endotoxin-targeting devices, such as Toraymyxin [17]. The mHLA-DR is a marker of immune competence, with low expression indicating immunosuppression and increased risk of secondary infections [8]. However, its measurement requires flow cytometry and is not yet standardized for routine clinical use. Cell-free DNA (cfDNA), released during cellular damage, has also emerged as a potential marker of disease severity and may correlate with cytokine levels [23]. Despite their promise, these biomarkers are not yet validated for guiding timing, duration, or repetition of adsorption therapy, and further studies are needed to establish their clinical utility. Second, there is a need for the development of more selective and customizable devices. Currently, existing devices often remove a broad range of molecules, which may not always be beneficial. Future devices could be designed to selectively target specific cytokines or pathogens, minimizing unintended consequences such as the removal of protective mediators. Selective adsorption aims to remove harmful mediators such as IL-6, TNF-α, and IL-1β, while sparing beneficial molecules like IL-10, which has anti-inflammatory properties, and growth factors such as VEGF and Epidermal Growth Factor (EGF), which support tissue repair and immune resolution [4,5]. Current non-selective devices may inadvertently remove these protective mediators, potentially impairing recovery. Future technologies may incorporate ligand-specific surfaces or molecular sieving strategies to enhance selectivity and minimize unintended immunomodulation. Additionally, combining cytokine adsorption with immunomodulatory therapies, e.g., checkpoint inhibitors, may enhance the therapeutic effect by reducing inflammation and modulating the immune system to better fight the infection. Furthermore, large-scale clinical trials with well-defined endpoints are essential to validate the benefits of extracorporeal cytokine adsorption. These trials should aim to clarify its role in reducing mortality and improving patient outcomes across different septic phenotypes, providing more robust evidence for clinical adoption. In addition, future research should investigate the pharmacokinetic interactions between cytokine adsorption devices and concomitant pharmacological therapies. Non-selective adsorption systems, such as CytoSorb, may inadvertently remove therapeutic agents, including antibiotics, corticosteroids, and immunomodulatory drugs. This could compromise treatment efficacy and contribute to subtherapeutic drug levels in critically ill patients. Although some devices like Seraph 100 have demonstrated minimal adsorption of antibiotics [20,84], further pharmacokinetic and pharmacodynamic studies are needed to quantify drug removal and develop evidence-based guidelines for safe co-administration [71].

Another key area for future research is the integration of sepsis phenotyping into the clinical decision-making process for extracorporeal cytokine adsorption. As sepsis encompasses a spectrum of immune responses (from hyperinflammation to profound immunosuppression) identifying patient subgroups based on immune profiles could help tailor the timing, intensity, and duration of adsorption therapies [4,8]. Biomarkers such as IL-6, CRP, and monocyte HLA-DR expression may serve as useful tools for stratification, although their routine clinical use remains limited [23]. Future clinical trials should incorporate immunophenotyping strategies to evaluate whether specific phenotypes can provide greater benefit (or potential harm) from cytokine removal. This personalized approach could represent a paradigm shift in the application of extracorporeal therapies in sepsis, moving from a one-size-fits-all model to a more targeted and effective intervention.

## 8. Conclusions

Extracorporeal cytokine adsorption has shown promise in sepsis management by reducing inflammatory mediators and improving organ function, particularly in severe sepsis, ARDS, and cardiogenic shock. While reductions in cytokine levels are consistent, the impact on mortality remains uncertain. Current evidence supports its use to reduce vasopressor requirements and enhance organ recovery, but further research is needed to confirm its mortality benefit. Future studies should focus on optimizing device selectivity, utilizing biomarkers for personalized therapy, and exploring combination treatments. Large-scale trials with clear endpoints and patient selection will be essential to define the role of extracorporeal cytokine adsorption in sepsis.

## Figures and Tables

**Table 1:** Comparative overview of extracorporeal blood purification devices. All devices are designed for single use only, according to the manufacturers’ specifications.

Device	Technology	Clinical Application	Regulatory Approval	Limitations/Side Effects	Recommended Treatment Duration
**CytoSorb®** (CytoSorbents Corps)	Hemadsorption 300 mL cartridge with porous polystyrene-divinyl-benzene polymer beads with a highly porous and biocompatible polyvinylpyrrolidone cover (~40,000 m^2^ surface area); adsorbs cytokines (5–60 kDa), bilirubin, myoglobin, DAMPs, PAMPs, certain drugs [67,68,69,70,71]	Septic shock, cytokine storm, ECMO, CRRT adjunct, drug intoxication [67,72,73,74].	CE Mark (EU), FDA EUA (USA, COVID-19 only), Approved in >70 countries	Non-selective adsorption may remove beneficial molecules and drugs. Coagulation system interaction- requires anticoagulation during use. Does not remove endotoxins efficiently compared to endotoxin-specific columns [40,71,75].	6–24 h per cartridge; may be used repeatedly in 24–72 h cycles
**Toraymyxin® (PMX-20R)** (Toray Industries, Inc.)	Antibiotic Polymyxin B immobilized on polystyrene fibres; targets endotoxins (LPS) [17,34].	Gram-negative sepsis with high EAA; reduces vasopressor need [76].	CE Mark, Japan PMDA approved; not FDA approved	Specific to endotoxins; no cytokine removal; benefit debated in some RCTs [77].	Typically 2 sessions of 2 hours each within 24 h
**Oxiris®** (Baxter)	Three-layer membrane structure: -AN69-based haemofilter: provides the primary renal support by diffusion and convection and adsorbs positively charged cytokines -Heparin grafting: reduce membrane thrombogenicity, minimize treatment interruptions, and support efficient CRRT -PEI coating: enhances hemocompatibility and enables the adsorption of negatively charged endotoxins (LPS) Combines cytokine, endotoxin removal + CRRT [22,78].	Sepsis with AKI; integrated with CRRT circuits (CVVH, CVVHDF) [78,79,80].	CE Mark (EU); FDA (USA, COVID-19 only)	Risk of clotting if anticoagulation inadequate; may require high blood flow rates [78].	Typically replaced every 12-24 h during CRRT sessions
**HA330/HA380** (Jafron Biomedical Co)	Resin-based hemadsorption (styrene-divinylbenzene copolymer); non-selective cytokine, bilirubin, tryptophan removal via hydrophobic interactions, Van der Waals forces, pore structure (wide range of pore sizes, e.g., 10 kDa to 60 kDa for HA380) [19,77].	Sepsis, SIRS, hyperinflammatory syndromes (common in China/Asia), integrated with CVVHDF [19,81,82].	CFDA (China); CE Mark (Europe); Not FDA approved	Non-selective; hypotension during use; limited high-quality data from Western trials [19,77].	2–4 h per session; 1–2.5 sessions depending on severity [77]
**Seraph® 100 Microbind®** (ExThera Medical Corporation)	Heparin-coated beads mimic endothelial glycocalyx (~40 m^2^ surface area); binds pathogens (bacteria, viruses, fungi) and toxins by electrostatic interaction simulating their natural binding site. Negligible clearance of most antibiotics [83,84,85].	Bloodstream infections (e.g., *S. aureus*), viral sepsis (e.g., COVID-19); pathogen clearance [86,87,88].	CE Mark, FDA EUA (USA)	Relevant initial adsorption of aminoglycosides. limited large-scale RCTs [85].	Up to 5 h per cartridge; max once daily; duration based on pathogen load

*Note:* AN69: Acrylonitrile and sodium methallylsulfonate copolymer; CE Mark: European Conformity marking (EU regulatory approval); CFDA: China Food and Drug Administration; CRRT: Continuous Renal Replacement Therapy; CVVH: Continuous Veno-Venous Hemofiltration; CVVHDF: Continuous Veno-Venous Hemodiafiltration; DAMPs: Damage-Associated Molecular Patterns; EAA: Endotoxin Activity Assay; ECMO: Extracorporeal Membrane Oxygenation; FDA: Food and Drug Administration; FDA EUA: U.S. Food and Drug Administration Emergency Use Authorization; LPS: Lipopolysaccharide; PAMPs: Pathogen-Associated Molecular Patterns; PEI: Polyethyleneimine; PMDA: Pharmaceuticals and Medical Devices Agency (Japan); SIRS: Systemic Inflammatory Response Syndrome.

## Data Availability

Not applicable.

## Abbreviations

AKI: Acute Kidney Injury; AN69: Acrylonitrile and sodium methallylsulfonate copolymer; ARDS: Acute Respiratory Distress Syndrome; CE Mark: European Conformity marking (EU regulatory approval); CFDA: China Food and Drug Administration; CLR: C-type Lectin Receptor; CRP: C-reactive protein; CRRT: Continuous Renal Replacement Therapy; CVVH: Continuous Veno-Venous Hemofiltration; CVVHDF: Continuous Veno-Venous Hemodiafiltration; CYSS: CytoSorb Therapy for Sepsis and Septic Shock; DAMPs: Damage-Associated Molecular Patterns; DIC: Disseminated Intravascular Coagulation; EAA: Endotoxin Activity Assay; ECMO: Extracorporeal Membrane Oxygenation; FDA: Food and Drug Administration; FDA EUA: U.S. Food and Drug Administration Emergency Use Authorization; IL: Interleukin; LPS: Lipopolysaccharide; MHC-II: Major Histocompatibility Complex Class II; mHLA-DR: Monocyte human leukocyte antigen-DR; NK: Natural Killer; NLR: Nod-like Receptor; PAMPs: Pathogen-Associated Molecular Patterns; PEI: Polyethyleneimine; PMDA: Pharmaceuticals and Medical Devices Agency (Japan); RIG: Retinoic Acid-Inducible Gene-I-Like Receptors; SIRS: Systemic Inflammatory Response Syndrome; sPD-L1: Soluble Programmed Death-Ligand 1; TLR: Toll-like Receptors; TNF-α: Tumor Necrosis Factor-α.
